# Social Defeat, Psychotic Symptoms, and Crime in Young Caribbean Immigrants to Rotterdam

**DOI:** 10.3389/fpsyt.2021.498096

**Published:** 2021-04-07

**Authors:** David J. Vinkers, Micha Van de Vorst, Hans W. Hoek, Jim Van Os

**Affiliations:** ^1^School for Mental Health and Neuroscience, Maastricht University, Maastricht, Netherlands; ^2^Psychiaters Maatschap Antillen, Willemstad, Curaçao; ^3^Parnassia Psychiatric Institute, The Hague, Netherlands; ^4^Department of Psychiatry, University of Groningen, University Medical Center Groningen, Groningen, Netherlands; ^5^Mailman School of Public Health, Columbia University, New York, NY, United States; ^6^Department of Psychiatry, UMC Utrecht Brain Centre, University Medical Centre Utrecht, Utrecht University, Utrecht, Netherlands; ^7^Department of Psychiatry and Neuropsychology, School for Mental Health and Neuroscience, Maastricht University Medical Center, Maastricht, Netherlands; ^8^Department of Psychosis Studies, Institute of Psychiatry, Psychology and Neuroscience, King's College London, London, United Kingdom

**Keywords:** social defeat, acculturation, psychosis, crime, Caribbean, immigrants

## Abstract

**Background:** The negative experience of being excluded from the majority group (social defeat) may be associated with psychosis in immigrants. The social defeat hypothesis is supported by the high frequency of perceived discrimination and acculturation problems in psychotic immigrants. In addition, social defeat may lead to crime through social problems such as unemployment, school dropout, a broken family structure, or psychotic symptoms.

**Methods:** We assessed the association between social defeat and acculturation on the one hand and broadly defined psychotic symptoms and crime on the other in Caribbean immigrants to Rotterdam who are aged 18–24 years. The municipality of Rotterdam provided data about Caribbean immigrants to Rotterdam. Acculturation, social defeat (perceived discrimination, sense of control, and evaluation of self and others), psychotic symptoms, and crime were assessed using online questionnaires.

**Results:** Social defeat was associated with psychotic symptoms in women (β = 0.614, *p* < 0.001). This relation applied particularly to the negative self-perception domain of social defeat. Acculturation was associated with neither social defeat nor psychotic symptoms or crime and did not mediate the association between social defeat and psychosis.

**Conclusion:** The social defeat hypothesis of psychosis may be gender-specific valid but does not extend to crime.

## Introduction

Immigration has been recognized as a risk factor for the development of psychosis since Ødegaard published his landmark study in 1932 (1). The findings of Ødegaard were replicated after World War II in Britain with the emergence of large-scale immigration (2–4). African-Caribbean immigrants were especially found to be at risk of developing psychosis (5–8). Studies in the Netherlands confirmed this increased risk of psychosis in immigrants (9–13). The relative risk of psychosis in immigrants is increased five times higher in immigrants with a dark skin color (14). Psychotic immigrants often feel discriminated, have lost their ties with their own culture, and struggle with social problems (15–19). Immigrants living in neighborhoods where they form a minority and have little support from ethnic peers are at the highest risk of a psychosis (20, 21). This observation was already made in the 1930's, when Faris and Dunham stated that “the extended isolation of the person with the role of an outcast” and “lack of sufficient self-confidence” were associated with psychosis in immigrants (22).

These findings led to the hypothesis that the increased incidence of psychosis in immigrants is associated with the experience of social defeat (23, 24). This social defeat hypothesis posits that the negative experience of being excluded from the majority group is the common denominator of major schizophrenia risk factors such as migration, urban upbringing, childhood trauma, and drug abuse (25).

Social defeat has also been linked to the high crime rate of Caribbean immigrants to Western European countries such as the United Kingdom and the Netherlands (26, 27). Caribbean immigrants have the highest crime rate of all population groups in the Netherlands with an annual conviction rate of 100 crimes per 1,000 persons (28). The crime rate on the Dutch Caribbean themselves is, in contrast, <6 per 1,000 persons (29). The high crime rate of Caribbean immigrants to the Netherlands has been related to socio-economic and cultural problems, such as school dropout, financial problems, a broken family structure, discrimination, and acculturation problems (30). In general, immigrants have lower crime rates, a phenomenon called the healthy immigrant effect (31). It is believed that the lower crime rate in immigrants is explained by strong social ties and more traditional values (32). Later generations of immigrants lose these traditional cultural values and become more adapted to their host country (33). A weakening of social bonds in immigrants (e.g., dropping out of school and having a lack of attachment to society) may lead to a low self-control and crime (34, 35). Social defeat may also increase crime rates in Caribbean immigrants through psychotic symptoms, as this is an independent risk factor for crime (36, 37). This is supported by the disproportionately high number of psychotic Caribbean immigrants in forensic psychiatric hospitals in Western Europe (38–41). Psychotic subjects may also be more likely to become victims of violence (42).

Hitherto, the relation between social defeat and psychosis and crime in immigrants has not been studied in a single study. Furthermore, most studies in this topic are case–control, which makes it difficult to disentangle the direction of the relationship. We examined if social defeat (perceived discrimination, sense of control, and evaluation of self and others) and acculturation were associated with psychotic symptoms and crime in young Caribbean immigrants to Rotterdam. We hypothesized that both social defeat and acculturation were associated with psychotic symptoms and crime. As there are indications for different pathways in male and female immigrants to the Netherlands (15), we analyzed these groups separately. We hypothesized furthermore that psychotic symptoms themselves were associated with crime.

## Methods

### Participants

Caribbean immigrants form a well-defined ethnic group with a significantly increased risk of psychotic symptoms and crime (9–13, 38–42). Rotterdam has the largest number of Caribbean immigrants in the Netherlands (28). Caribbean ethnicity was defined, in line with the Dutch Central Bureau of Statistics, as a person with at least one parent born in one of the former Dutch Antilles or Aruba (28, 29). In addition, participants were asked if they saw themselves as having a Caribbean ethnicity, a commonly used assessment of ethnic group in the Netherlands. The Municipality of Rotterdam provided addresses of all Caribbean immigrants to Rotterdam aged 18–24 years. Data pertaining to 2015, 2016, and the first half of 2017 were received quarterly. The sampling frame consisted of 2,388 persons. They were sent a written invitation to participate and were offered a 15-euro gift certificate for participation. In total, 64 participants enrolled and started with the online questionnaire. Eleven questionnaires were omitted because not all questions were answered. In total, 53 participants were included in the study.

### Questionnaires

#### Acculturation

The process of becoming part of a new culture is called acculturation. Acculturation is a dynamic characteristic that develops and changes over time (43). It reflects the degree in which the original culture is retained while adapting to the new one (44). Both a weak and a strong involvement in the original culture has been described as a risk factor for psychosis in immigrants (45, 46). For acculturation, the Ethnic and National Identity questionnaire (12 items) was used. This questionnaire has response options ranging from “strongly disagree” (1) to “strongly agree” (5). It assesses ethnic and national affirmation, sense of belonging, and feelings about being a national and ethnic group member. It has a sufficient reliability with a Cronbach alpha higher than 0.8 (44). Acculturation was assessed as Dutch and Antillean cultural acculturation scores, with higher scores indicating a higher degree of involvement in the Dutch or Antillean culture.

### Social Defeat

Social defeat is defined as the negative experience of being excluded from the majority group (14–16, 23, 24). We assessed social defeat as the total sum score of sense of control and evaluation of self and others minus perceived discrimination. Higher scores on social defeat indicate more experience of social defeat (more discrimination, less control, and negative evaluation of self and others). The subfactors were assessed by the questionnaires from the International Comparative Study of Ethnocultural Youth (ICSEY) (44, 46) and the Brief Core Schema Scales (BCSS) (47). The ICSEY is an international project studying the adaptation and integration of immigrant juveniles. The ICSEY questionnaires have been validated for use in the Netherlands (44). The ICSEY perceived discrimination subscale consists of five items about the experience of being treated negatively or threatened, with responses ranging from “strongly disagree” (score 1) to “strongly agree” (score 5) with a reliability Cronbach alpha of 0.83 (46). The ICSEY sense of control subscale consists of six items and assesses the feeling of being in control (e.g., “What happens in the future mostly depends on me”), with responses ranging from “strongly disagree” (score 1) to “strongly agree” (score 5).

The BCSS is a 24-item self-report assessment of schemata concerning self and others (47). It has good psychometric properties with an internal consistency of (alpha) 0.78 for evaluation of self and 0.88 for the evaluation of others in non-clinical samples and a median item correlation of 0.78 (47). The scale evaluates four dimensions of self and others: negative-self (six items), positive-self (six items), negative-other (six items), and positive-other (six items). The scale was recoded to give scores about negative evaluation of self and others. The Dutch translation used in the international EUGEI study was used with a 5-point Likert scale with scores ranging from 6 up to 30 (48).

### Broadly Defined Psychotic Psychopathology

Psychotic symptoms were assessed with the Community Assessment of Psychic Experiences (CAPE-42) (49, 50). The CAPE-42 is a validated 42-item self-report questionnaire of psychotic symptoms (51). It has good reliability and validity with intraclass correlation coefficients of 0.62–0.64 for the positive, negative, and depressive dimensions (49). Each item explores the frequency of the experience on a 4-point scale of “never” (score 1) to “nearly always” (score 4) and the degree of distress associated with this experience on a 4-point scale of “not distressed” (score 1) to “very distressed” (score 4). The CAPE-42 has 20 items of positive psychotic symptoms, 14 items of negative experiences, and 8 items of depressive experiences (49–51). Positive symptoms reflect an excess or distortion of normal functions, e.g., delusions, hallucinations, and disorganized thought. Negative symptoms reflect an absence or loss of normal abilities, such as flat or blunted affect and emotion, lack of motivation, and poverty of speech. Higher scores indicate more symptoms on each scale.

### Crime

Delinquent behavior was assessed using a 25-item self-report questionnaire, validated in the Netherlands (52). The questionnaire is sensitive for delinquent behavior in juvenile Caribbeans (53). Earlier research showed a satisfactory reliability by comparing the self-reported crime scores with data from the police (54). The questions are about minor and frequently occurring offenses, e.g., fare dodging in public transport, vandalism, and shoplifting, but also for serious and less frequent ones, e.g., burglary, robbery, and hurting someone with a weapon. The offenses include property offenses, vandalism, and violent offenses. A total score was calculated for all crimes. In addition, participants were asked if they ever were arrested, taken to prison, or convicted, but all participants denied this question.

### Procedure

Those who agreed to participate were asked to fill in a consent form and to provide their contact information (email address and telephone number). Participants subsequently received an email containing a link to the online questionnaire. The online questionnaire consisted of ~157 questions and took about 30 min to complete. The questionnaire screened acculturation, social defeat, psychosis, and crime. The standing Medical Ethical Committee of Maastricht University Hospital approved the study.

### Data Analysis

We used Stata to analyze the data. The association between psychotic psychopathology (outcome factor) and social defeat, acculturation for the Dutch and Antillean culture, gender, and level of education was assessed with multivariable regression analysis. Level of education was classified as low in case of only lower general secondary education. Gender differences and differences in educational level for associations between social defeat and broad psychotic psychopathology were analyzed with multivariable regression. Also, multivariable regression was used to analyze the association between subfactors of social defeat and broad psychotic psychopathology. A logistic regression analysis was used to analyze the association between self-reported crime, given its zero-inflated distribution, as outcome factor and social defeat, acculturation for the Dutch and Antillean culture, gender, and level of education as independent variables. The Jarque–Bera test was used to assess normality for broad psychotic psychopathology. Heteroscedasticity was analyzed using the Breusch–Pagan/Cook–Weisberg test. Lastly, structural equation modeling was used to analyze mediation effects on the associations as assessed in the described multivariable and logistic regression analysis.

## Results

Table 1 shows the demographic characteristics of the participants. Female participants had a higher score on the CAPE-42 regarding the total, negative, and depressive symptom scores (*t*-test, *p* < 0.05).

**Table 1:** Demographics of 53 young Caribbean immigrants to Rotterdam.

	**Male,** ***n* = 18 (34.0%)**	**Female,** ***n* = 35 (66.0%)**
**Place of growing up**		
Curaçao	12 (66.7%)	14 (40.0%)
Bonaire	2 (11.1%)	2 (5.7%)
Aruba	3 (16.7%)	12 (34.3%)
Netherlands	1 (5.6%)	7 (20.0%)
**Level of education**		
Lower general secondary education	10 (55.6%)	18 (51.4%)
Higher general secondary education	8 (44.4%)	17 (48.6%)
**Lived in the Netherlands before**		
Yes	5 (27.8%)	8 (22.9%)
No	13 (72.2%)	27 (77.1%)
Years lived in the Netherlands	0.88 (0.67)	1.28 (1.17)
Social defeat	17.2 (15.3)	18.5 (9.5)
Negative perception of others	4.8 (5.7)	6.3 (4.4)
Negative perception of self	14.9 (8.1)	15.5 (5.0)
No feeling of control	9.5 (2.5)	8.4 (2.6)
Discrimination	12.1 (6.0)	11.7 (4.1)
Psychotic psychopathology (CAPE-42)	56.8 (6.8)	62.3 (9.8)
Acculturation	35.9 (6.2)	33.7 (7.1)
Dutch culture	11.8 (3.4)	10.6 (4.4)
Antillean culture	24.1 (4.5)	23.1 (5.1)
Self-reported crime	0.78 (1.06)	1.09 (1.38)

*Continuous measurements are presented as mean with standard deviation*.

Table 2 shows the results of the multiple regression analysis for self-reported psychotic symptoms. The results of the regression indicated that the five predictors explained 40.5% of the variance [*R*^2^ = 0.405, *F*_(6,47)_ = 6.41, *p* < 0.01). Psychotic psychopathology was predicted by gender (β = −1.13, *p* < 0.01) and social defeat (β = 1.52, *p* < 0.001). Acculturation and education were not significantly associated with psychotic symptoms. The association between social defeat and psychotic psychopathology [analyzed in a multivariable regression, *R*^2^ = 0.768, *F*_(5,48)_ = 39.68, *p* < 0.001] was negative for male participants (β = −0.207, *p* < 0.05) and positive for female participants (β = 0.614, *p* < 0.001). We found no significant association with acculturation, level of education, length of stay in the Netherlands, or earlier stay in the Netherlands and neighborhood on social defeat, psychotic symptoms, or crime.

**Table 2:** Predictive factors for psychotic psychopathology in 53 young Caribbean immigrants to Rotterdam.

**Independent variable**	***ß***	***p***	***r***
Gender	−1.13	0.001^†^	0.286
Level of education	−0.01	0.927	0.039
Social defeat	1.52	0.000^†^	0.479
Antillean culture acculturation	−0.13	0.280	−0.022
Dutch culture acculturation	−0.05	0.670	−0.024

† *Significant for α = 0.05*.

The association between subscales of social defeat (self-control, discrimination, perception of others, and self-perception) and psychotic symptomatology was also tested in a multiple regression analysis. The direct effect of social defeat on psychotic symptoms was 0.35 (*z* = 3.52, 95% CI = 0.16–0.55), and the indirect effect mediated by acculturation was 0.00 (*z* = −0.26, 95% CI = −0.02–0.02). It was found that self-perception significantly predicted psychotic psychopathology (β = −0.301, *p* < 0.04). For all multivariable regression analysis, no assumptions for normality or heteroscedasticity were violated (Jarque–Bera and Breusch–Pagan/Cook–Weisberg, both *p* > 0.05). To avoid multicollinearity, predictive factors with a Pearson correlation bigger than 0.30 were omitted, which was the case for perceived discrimination. A log linear regression analysis was used to test if social defeat, gender, education, and acculturation predicted crime. The likelihood ratio of this model was χ^2^(5, *N* = 53) = −35.79, *p* = 0.88, indicating no associations were found. Structural equation modeling showed no evidence that the effect of social defeat on psychotic psychopathology was mediated through acculturation.

## Discussion

This study shows that social defeat is associated with psychotic symptoms in young female Caribbean immigrants to the Netherlands. Psychosis has in general a later age of onset and probably a more favorable course in women (55). Subclinical psychotic symptoms are, however, more frequent in young female adults, which is in line with our findings (56). In international studies, there are no significant differences between male and female immigrants regarding the incidence of psychosis (57). In contrast, the risk of psychosis in immigrants to the Netherlands is clearly increased for male as compared to female immigrants (15). This is especially true for Moroccan immigrants to the Netherlands (with a 5-fold increased risk of psychosis in males), but also for Caribbean immigrants (15). The gender gap may be related to more substance abuse, discrimination, and an unfulfilled wish to achieve in the new society (15, 45). We could, from our data, not confirm or refute this hypothesis. We found, however, no difference in experienced discrimination between male and female participants.

As the participants have lived in the Netherlands for only a short time (~1 year on average), it is difficult to disentangle the temporal relation between social defeat and psychotic symptoms in female participants in this cross-sectional study. This relationship was, however, independent of acculturation (regarding both the Dutch and the Antillean cultures), neighborhood, or level of education. Although there are indications that psychosis in immigrants is related to cultural marginalization and a low ethnic density of the neighborhood (58), their temporal relationship remains unclear.

We found a negative association between social defeat and psychosis in male immigrants. It is possible that this is a false-positive finding, especially regarding the significant relationship in the other direction in female immigrants. On the other hand, male subjects with psychotic symptoms may defensively deny any manifestations of social defeat. It is also possible that our assessment may be too early for male participants to establish an association between social defeat and psychotic symptoms. Other studies show that the time between migration and onset of psychosis is typically several years (56). Hollander found, for example, that the time from migration to first diagnosis was around 3 years for non-refugee migrants (58). Other explanations for this finding may be that the group of male participants was too small or less accurate in the online reporting of social defeat and psychosis variables. The concept of social defeat may also require further clarification and further development, as the classification of social defeat factors may be too crude and too general (59).

Female participants reported more psychotic symptoms (especially depressive and negative symptoms) than did male participants in our study. The reported scores of psychotic symptoms and a negative perception of self were relatively high in both male and female participants as compared to non-immigrant samples from the general population (44–46, 49–51). The relation between social defeat and psychotic symptoms was significantly associated with a negative self-perception, but not with the other subscales of social defeat (discrimination, low self-control, and negative perception of others). This may suggest that internal factors are more important than external factors in the relation between social defeat and psychosis. Other studies show that social defeat may be associated with depressive and negative psychotic symptoms through negative schemas and loneliness (60).

Acculturation, level of education, neighborhood, or length of stay in the Netherlands were not associated with social defeat, psychotic symptoms, or crime. Although we did not have a large study group and therefore had limited power, there was sufficient sensitivity to demonstrate a significant association between social defeat and psychosis. Other limitations were that crime was based on self-report and that most participants were female, a population at relatively lower risk for delinquency.

Another important finding of this study is that crime in young Caribbean immigrants was not associated with social defeat, acculturation, or psychotic symptoms. The crime rate of this group in the Netherlands is high, with an annual conviction rate of 100 crimes per 1,000 persons (28). This has been related to socio-economic and cultural problems, among others discrimination and acculturation problems (30). We could, however, not establish this association in this group of Caribbean immigrants. Our participants had to participate in the study themselves and may be therefore biased toward less crime. Immigrants have lower crime rates, a phenomenon called the healthy immigrant effect (31). We did not find a relation with acculturation or social defeat. There are possibly other factors explaining the high crime rate in Caribbean immigrants to the Netherlands. In line with this, we found in an earlier study that psychosis did not explain the increased crime rate of Caribbean immigrants to the Netherlands (38).

In conclusion, we found that social defeat is associated with psychotic symptoms in young female Caribbean immigrants to the Netherlands. This was especially related to the internal factor of negative self-perception. Our findings are in line with the social defeat hypothesis, which relates social defeat to psychosis in immigrants (15–19, 23–25). More research is needed to assess the temporal association between social defeat and psychotic symptoms. There may be other mechanisms explaining psychosis in male and female immigrants. Crime in this group was not associated with social defeat, acculturation, or psychotic symptoms.

## Data Availability Statement

The datasets generated for this study are available on request from the corresponding author.

## Ethics Statement

The studies involving human participants were reviewed and approved by the METC azM/UM, the medical ethical review committee of Maastricht University, the Netherlands (NL48702.068.14 / METC143017). The patients/participants provided their written informed consent to participate in this study.

## Author Contributions

DV, HH, and JO conceptualized the study. DV and MV analyzed the data and drafted the manuscript. MV contributed to the analysis and interpretation of the data. All author critically revised the manuscript and contributed specific expertise.

## Conflict of Interest

The authors declare that the research was conducted in the absence of any commercial or financial relationships that could be construed as a potential conflict of interest.
